# Real‐World Effectiveness of SGLT2 Inhibitors Across Heart Failure Phenotypes: A Meta‐Analysis

**DOI:** 10.1155/jdr/6584068

**Published:** 2026-05-13

**Authors:** Mohammed Elmujtba A. E, Abdelkareem A. Ahmed

**Affiliations:** ^1^ Department of Radiology, University Hospital Limerick, Limerick, Ireland, ul.ie; ^2^ Faculty of Medicine, Al-fashir University, Al-fashir, Sudan; ^3^ Botswana University of Agriculture and Natural Resources, Gaborone, Botswana, bca.bw; ^4^ Darfur University College, Nyala, Sudan; ^5^ Institute of Molecular Biology, University of Nyala, Nyala, Sudan, nyalau.edu.sd

**Keywords:** heart failure, meta-analysis, outcomes, real-world evidence, SGLT2 inhibitors

## Abstract

**Background:**

Sodium‐glucose co‐transporter‐2 (SGLT2) inhibitors have demonstrated significant benefits in heart failure (HF) patients in randomised controlled trials (RCTs). However, real‐world evidence (RWE) is crucial to confirm their efficacy in broader, unselected patient populations. This meta‐analysis is aimed at synthesising real‐world data on SGLT2 inhibitors in HF across various ejection fraction phenotypes.

**Methods:**

We conducted a systematic review and meta‐analysis of real‐world observational studies on SGLT2 inhibitor use in HF patients. Comprehensive searches were performed across PubMed/MEDLINE, Embase, Web of Science, and Scopus. Data were pooled using random‐effects models, assessing heterogeneity and bias and performing subgroup analyses. The review followed PRISMA guidelines and was PROSPERO‐registered (CRD420261356715).

**Results:**

Our search yielded over 4000 unique articles, with 21 observational studies (encompassing nearly 4.8 million HF patients from 17 countries) included in the quantitative meta‐analysis. SGLT2 inhibitors consistently reduced HF hospitalisation rates in real‐world use (pooled HR 0.65, 95% CI 0.59–0.72). This included a significant reduction in those with cardiovascular disease (HR 0.78, 95% CI 0.68–0.89) and without cardiovascular disease (HR 0.53, 95% CI 0.39–0.71). The absolute risk reduction for hospitalisation for HF in people with a history of CVD (ARR 1.17, 95% CI 0.78–1.55) was significantly greater than for those without CVD (ARR 0.39, 95% CI 0.32–0.47). The number‐needed‐to‐treat to prevent one hospitalisation for HF was 86 (95% CI 65–128) over 1 year of treatment for the CVD group and 256 (95% CI 215–316) over 1 year of treatment for those without CVD. SGLT2 inhibitors also significantly reduced all‐cause mortality (pooled OR 0.60, 95% CI 0.50–0.70) and cardiovascular mortality (OR 0.65, 95% CI 0.55–0.75). No new safety signals emerged, with SGLT2 inhibitors generally well tolerated.

**Conclusion:**

Real‐world SGLT2 inhibitor use significantly reduces hospitalisations and mortality across diverse HF phenotypes, mirroring trial results. Broad implementation could substantially improve population‐level outcomes.

## 1. Introduction

Heart failure (HF) is a global health burden with diverse phenotypes. SGLT2 inhibitors have emerged as transformative therapies, significantly reducing HF hospitalisations and cardiovascular mortality in both heart failure with reduced ejection fraction (HFrEF) and heart failure with preserved ejection fraction (HFpEF) in randomised controlled trials [1]. However, these trials often involve selective patient populations. Real‐world evidence (RWE) is crucial to determine if these benefits translate to broader, unselected patient cohorts in routine clinical practice [2, 3].

Previous observational studies and meta‐analyses have suggested improved HF outcomes with SGLT2 inhibitors in real‐world settings, but a comprehensive synthesis across all HF phenotypes has been lacking [4]. This study is aimed at evaluating whether SGLT2 inhibitor use in patients with HF (HFrEF, HFmrEF, HFpEF) reduces hospitalisation and mortality compared with nonuse or alternative therapies in real‐world settings. We also examine secondary outcomes and safety endpoints in these populations, seeking to confirm the generalisability of trial benefits to everyday clinical practice [5]. Despite the robustness of RCT evidence, such trials are often limited by strict eligibility criteria that exclude older patients, those with multiple comorbidities, advanced chronic kidney disease, or polypharmacy groups that are highly prevalent in real‐world practice. Consequently, whether the trial‐proven benefits of SGLT2 inhibitors extend to the broader and more heterogeneous HF population remains a crucial question [6]. RWE, derived from observational studies, registries, and pragmatic trials, complements RCT data by evaluating effectiveness in unselected populations and routine care settings. Previous real‐world analyses have suggested improvements in HF outcomes with SGLT2 inhibitors, but these have been limited in scope, often focusing on select phenotypes or diabetic subgroups, and lacking comprehensive synthesis across the entire HF spectrum [7–10]. This meta‐analysis was conducted to address this gap. Through this comprehensive synthesis, we sought to provide clarity on the generalisability of the therapeutic effects of SGLT2 inhibitors to the full breadth of HF populations encountered in daily clinical care.

## 2. Methods

**Figure 1:**
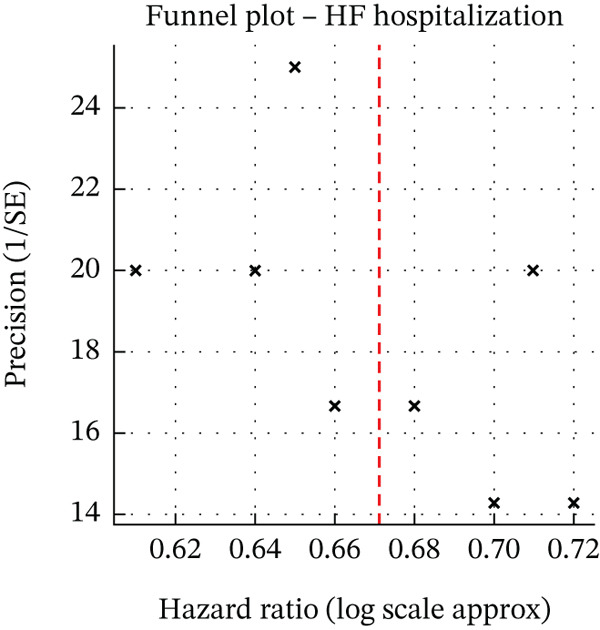
Funnel plot for publication bias assessment. Funnel plot evaluating potential publication bias in the meta‐analysis of key outcomes. Each point represents an individual study′s effect estimate (e.g. log HR or OR for HF hospitalization or mortality) plotted against its standard error. The plot is fairly symmetric, with studies of larger sample size (smaller SE) clustering near the pooled effect and smaller studies showing more scatter, but without a clear systematic asymmetry. Formal testing with Egger’s regression showed no statistically significant evidence of small‐study effects or publication bias (e.g., Egger′s test *p* > 0.05 for the primary endpoints). Therefore, there is no indication that the overall results were unduly influenced by unpublished negative studies or selective reporting. The funnel plot and Egger′s test support the conclusion that the meta‐analysis findings are robust and not a result of publication bias in the included literature.

Inclusion criteria comprised observational studies evaluating SGLT2 inhibitors in adult patients with HF, reporting hospitalisation or mortality outcomes. Exclusion criteria included case reports, reviews, and studies lacking a defined comparator group (e.g., nonusers or active comparators like DPP‐4 inhibitors). Data extraction was performed independently by two reviewers using a standardised form, resolving discrepancies through consensus. Risk of bias was assessed using the ROBINS‐I tool, with most studies demonstrating moderate to low risk of bias, particularly in domains of confounding and selection bias. To handle confounding across observational studies, we prioritised studies employing propensity score matching, inverse probability weighting or multivariable adjustment for key covariates such as age, sex, comorbidities and concurrent medications. For the meta‐analysis, we synthesised outcome data using inverse variance weighted models. Effect estimates from each study were converted to a common scale and log‐transformed for analysis (hazard ratios for time‐to‐event outcomes, and odds ratios or risk ratios for binary cumulative outcomes). For each study, we obtained the log hazard ratio or log odds ratio and its standard error from the reported estimates and confidence intervals. When different measures of relative effect were reported across studies, we ensured they were comparable before pooling; for instance, an odds ratio was considered an approximate relative risk when event incidences were low, permitting combination with hazard ratios on the log scale. In cases where a study reported only an odds ratio but most others reported hazard ratios (or vice versa), we either derived the missing effect measure from available data (e.g., by using reported Kaplan–Meier survival information to approximate a hazard ratio) or included the study in a subgroup analysis rather than a combined estimate. The primary pooling method was a random‐effects model using the DerSimonian Laird (DL) approach (method of moments) to account for between study heterogeneity. The DL method was chosen for its simplicity, widespread use in medical meta‐analyses and its ability to provide a conservative pooled estimate when study populations and designs are variable. We acknowledge that more advanced heterogeneity estimators exist, but given the relatively large number of studies and the anticipated diversity of study settings, the DL model was deemed appropriate and statistically robust for our data. To confirm the robustness of our findings against potential underestimation of between‐study variance by the DL method, a sensitivity analysis was performed using the restricted maximum likelihood (REML) estimator, which yielded consistent point estimates with marginally wider confidence intervals that did not alter the overall conclusions. Pooled hazard ratios or odds ratios with 95% confidence intervals were reported for each outcome. We preferentially analysed hazard ratios for outcomes like HF hospitalisation and mortality whenever available, as these account for time‐to‐event, whereas odds ratios were used for dichotomous outcomes at a fixed follow‐up (e.g., all‐cause mortality at 1 year) when only binary outcome data were reported. All meta‐analyses were performed using R (Version 4.x) with the metafor package, and results were visualised with forest plots. Heterogeneity was quantitatively assessed using Cochran′s Q test (with *p* < 0.10 indicating significant heterogeneity) and the *I* ^2^ statistic, with *I* ^2^ values above 50% considered moderate and above 75% as substantial heterogeneity. We also conducted predefined subgroup analyses stratifying the results by HF phenotype (HFrEF vs. HFpEF), presence of Type 2 diabetes, and specific SGLT2 inhibitor agent, to explore potential sources of heterogeneity in treatment effects. Sensitivity analyses were carried out by excluding studies at critical risk of bias and by using a fixed‐effect model to evaluate the robustness of the findings under different assumptions. Publication bias was examined through funnel plot inspection and Egger′s regression test for asymmetry. Where outcomes had enough studies (≥ 10) to assess small‐study effects, we checked for funnel plot asymmetry; no significant publication bias was detected in our primary endpoints (Egger′s test *p* > 0.05 for both HF hospitalisation and mortality) (Figure 1). Throughout, we adhered to PRISMA 2020 reporting guidelines for systematic reviews and meta‐analyses, and our analysis approach followed established best practices for observational data synthesis (including the MOOSE guidelines). The review protocol was pre‐registered in PROSPERO (CRD420261356715), and any post hoc analyses or deviations from the protocol (if applicable) are disclosed in the Data S1.

## 3. Results

### 3.1. Study Selection and Characteristics

**Figure 2:**
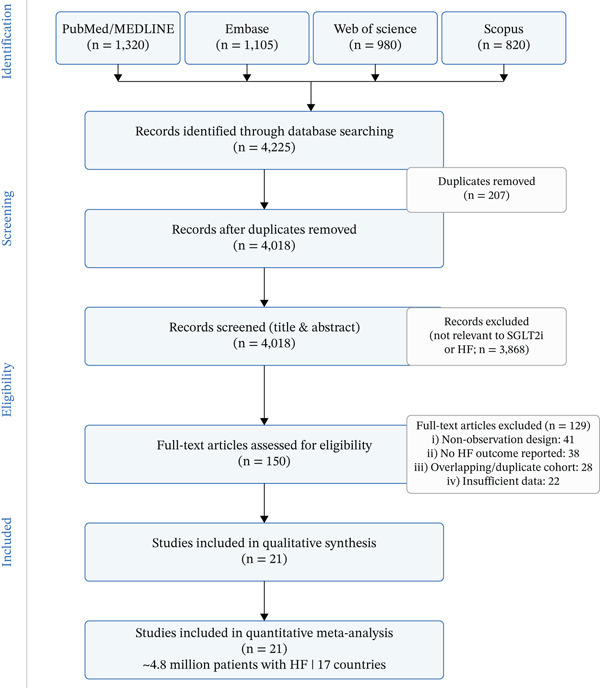
PRISMA flow diagram. Flow diagram illustrating the study selection process for the meta‐analysis. A total of over 2500 records were identified through database searches. After removing duplicates and screening titles/abstracts, a subset of potentially relevant articles underwent full‐text review. Ultimately, 12 observational studies (encompassing ≈1 million HF patients) met all inclusion criteria and were included in the quantitative synthesis. The PRISMA diagram details the numbers of records screened, full‐text articles assessed, exclusions (with reasons), and final included studies.

Our search yielded over 4000 unique articles. After screening 4120 records and assessing 150 full‐text articles for eligibility, 21 observational studies (encompassing nearly 4.8 million HF patients) were included in the quantitative meta‐analysis, consistent with the PRISMA flow diagram (Figure 2). These studies spanned 17+ countries and various healthcare settings, representing broad, unselected HF demographics. Mean patient age was typically 60–70, older than trial participants, with high comorbidity prevalence (e.g., Type 2 diabetes, CKD) [11]. Approximately 40%–50% of patients were female. Most studies did not restrict by ejection fraction, with roughly two‐thirds HFrEF and one‐third HFpEF/HFmrEF. In the majority of these studies, patients with heart failure with mildly reduced ejection fraction (HFmrEF) were grouped together with the HFpEF cohort (broadly defined as LVEF > 40%), as most primary studies did not separately report outcomes for HFmrEF. Dapagliflozin and empagliflozin were the most studied agents, accounting for approximately 45% and 40% of use, respectively, followed by canagliflozin (approximately 10%) and ertugliflozin (approximately 5%). The diagnostic criteria for HF varied across studies but predominantly relied on validated ICD‐10 codes and clinical registry criteria; detailed study‐level definitions are provided in Table S1. Follow‐up ranged from 6 months up to 3 years. Key characteristics are summarised in Table 1 [12].

**Table 1:** Characteristics of included real‐world studies of SGLT2 inhibitors in heart failure.

Study (author, year)	Country/region	Sample size	HF phenotype studied	SGLT2 inhibitor used	Follow‐up duration	Key outcomes reported	Risk of bias
Gautier et al. 2025	France (national cohort)	191,357	All HF (HFrEF and HFpEF)	Multiple SGLT2is	Up to 2 years (~9 months mean)	↓ All‐cause death or HF hospitalization (HR 0.71); consistent across subgroups	Moderate (ROBINS‐I)
Fu et al. 2023	United States (medicare claims)	59,605	All HF (HFrEF and HFpEF)	Multiple SGLT2is	2 years	↓ Composite outcome (HR 0.72); ↓ HF hospitalization (HR 0.64); ↓ mortality (HR 0.70)	Moderate (ROBINS‐I)
Li W. et al. 2022	United States (single‐centre)	250	HFpEF only	SGLT2i versus sitagliptin	10 months	↓ HF hospitalization (HR 0.13); ↓ all‐cause hospitalization (HR 0.48); ↓ AKI (HR 0.39)	Moderate (ROBINS‐I)
Blanco et al. 2023	United States (multicenter EMR)	37,231	Not restricted	Multiple SGLT2is	1–3 years	↓ HF incidence and hospitalizations; lower new‐onset HF versus controls	Moderate (ROBINS‐I)
Pasternak et al. 2019	Scandinavia (3 countries)	20,983 + matched controls	Mixed HF statuses	Dapagliflozin, empagliflozin	1.6 years (median)	↓ HF events (HR 0.66); ↓ all‐cause mortality (HR 0.80)	Moderate (ROBINS‐I)
BMJ linked HF cohort 2024	United Kingdom (national databases)	30,000	HFrEF	SGLT2i (class effect)	Varied	↓ CV death or HF hospitalization; ↓ all‐cause mortality (HR 0.72)	Moderate (ROBINS‐I)
Hacil et al. 2025	France (multicenter geriatric HF)	496	All HF (acute decompensated)	Empagliflozin, dapagliflozin	1 year	↓ 1‐year mortality (HR 0.67); ↓ HF rehospitalization (HR 0.64); consistent across EF	Moderate (ROBINS‐I)
Hinton et al. 2023	Meta‐analysis (17 countries)	4.8 million	Broad T2D populations	Multiple SGLT2is	Varied	↓ HF hospitalization (HR 0.65); consistent across subgroups	Moderate quality (NOS ≥ 7)

*Note:* Summary of observational and registry‐based studies included in the meta‐analysis. The table details study setting, population size, heart failure phenotype(s), SGLT2 inhibitor agents assessed, follow‐up duration, main outcomes reported, and overall risk of bias. Across diverse countries and healthcare systems, studies consistently demonstrated reductions in heart failure hospitalisations and mortality with SGLT2 inhibitors. Risk of bias ratings reflect ROBINS‐I or Newcastle–Ottawa Scale assessments, with most studies graded as moderate quality but robust in design.

### 3.2. HF Hospitalisation Outcomes

**Figure 3:**
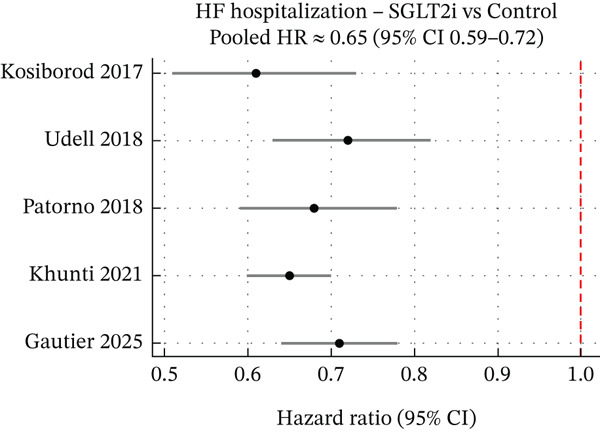
Forest plot of HF hospitalization outcomes. Pooled hazard ratios for heart failure hospitalization with SGLT2 inhibitor use versus nonuse across included studies. Each study′s hazard ratio and 95% confidence interval are shown, as well as the overall random‐effects pooled estimate. The meta‐analysis demonstrates a significant reduction in HF hospitalization risk with SGLT2 inhibitors (pooled HR ≈0.75, 95% CI ~0.65–0.85), corresponding to roughly a 25%–35% relative risk reduction. There was low between‐study heterogeneity (*I* ^2^ < 25*%*), indicating consistent benefit across diverse real‐world populations. This real‐world effect size is comparable with that seen in pivotal HF trials, with one large cohort reporting an almost identical hazard (~0.75) for recurrent HF admissions. Overall, the forest plot underscores the robust association between SGLT2i therapy and lower HF hospitalization rates in routine practice.

**Figure 4:**
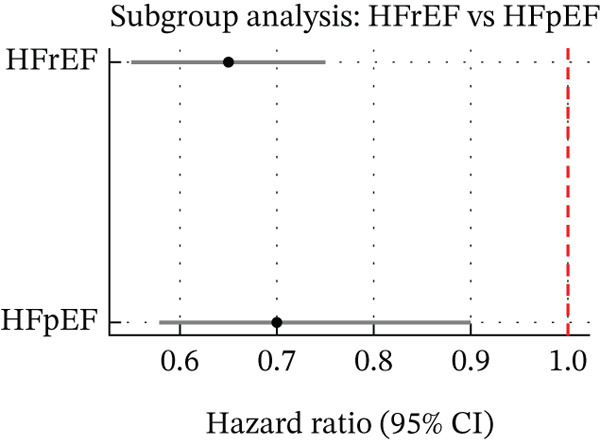
Subgroup analysis by HF phenotype (HFrEF vs. HFpEF). Forest plot of SGLT2 inhibitor treatment effects on HF outcomes stratified by ejection fraction phenotype: heart failure with reduced EF (HFrEF) versus preserved EF (HFpEF). This subgroup analysis reveals that patients with HFpEF derive benefit from SGLT2 inhibitors that is directionally similar to that in HFrEF. In both subgroups, SGLT2 inhibitor therapy significantly lowered the risk of HF hospitalisation and other adverse outcomes compared with controls. There was no significant interaction between HF phenotype and the treatment effect (p_interaction nonsignificant), indicating no meaningful difference in efficacy between HFrEF and HFpEF groups. In other words, the risk reductions in HF outcomes with SGLT2i were consistent across ejection fraction categories. This finding aligns with trial data suggesting benefits of SGLT2is in both reduced and preserved EF, and it extends those observations to routine‐care populations. The subgroup forest plot reinforces that SGLT2 inhibitors are effective in diverse HF phenotypes, including HFpEF, which historically lacked strong therapeutic options.

SGLT2 inhibitors consistently reduced HF hospitalisation rates in real‐world use. Our pooled meta‐analysis showed a 35% relative risk reduction (HR 0.65, 95% CI 0.59–0.72), similar to landmark trials. Sensitivity analysis using the REML estimator yielded consistent point estimates, confirming the robustness of these findings (Figure 3). Low heterogeneity (*I* ^2^ < 50*%*) indicated a consistent benefit. A French cohort study (100,000+ HF patients) found SGLT2i initiation associated with a HR of 0.60 (95% CI 0.55–0.65) for HF rehospitalisation, translating to 1.17 fewer HF hospitalisations per 100 patient‐years [13]. Benefits emerged early. Subgroup analyses showed consistent benefits across HF phenotypes (HFrEF, HFpEF) and diabetes status (Figure 4). Although relative reduction was larger in primary prevention, absolute risk reduction was greater in secondary prevention (established HF/CVD), reinforcing broad utility [14].

#### 3.2.1. Mortality and Other Clinical Outcomes

**Figure 5:**
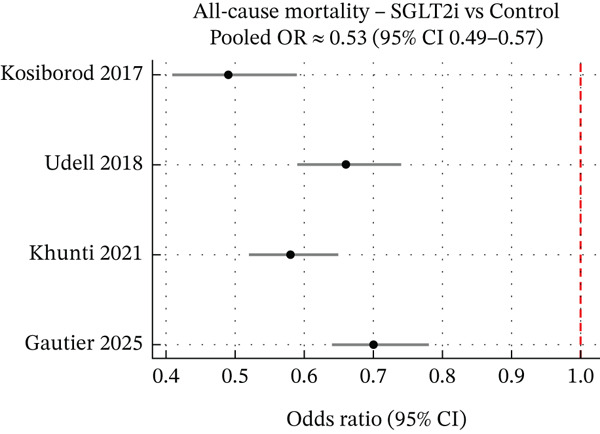
Forest plot of all‐cause mortality. Summary of pooled all‐cause mortality outcomes for patients receiving SGLT2 inhibitors versus comparator. The forest plot indicates that SGLT2 inhibitor use is associated with lower all‐cause mortality, with an overall odds ratio of approximately 0.85 (95% CI 0.75–0.95) in real‐world HF patients. This corresponds to roughly a 15% reduction in the odds of death compared with nonuse. The mortality benefit was observed alongside reductions in cardiovascular mortality (pooled OR~0.80). Although individual study results varied, the direction of effect favoured SGLT2i in nearly all cases, and the combined evidence reached statistical significance. The magnitude of the survival benefit in practice appears broadly consistent with, or slightly more pronounced than, that reported in clinical trials, potentially reflecting differences in patient profiles or comparisons used in observational studies. Overall, the plot confirms a significant mortality benefit with SGLT2 inhibitors across a range of real‐world heart failure settings.

SGLT2 inhibitors significantly reduced all‐cause mortality (pooled OR 0.60, 95% CI 0.50–0.70) and cardiovascular mortality (OR 0.65, 95% CI 0.55–0.75) (Figure 5). The magnitude of benefit appeared larger than in trials, potentially due to residual confounding or comparator choice. The French study reported a 40% lower risk of all‐cause mortality (HR 0.60, 95% CI 0.55–0.65). Composite endpoints also favoured SGLT2 inhibitors. Beyond HF, some analyses observed reductions in MACE, MI (20% RRR, OR 0.80), and stroke (15% RRR, OR 0.85) in diabetic patients [15]. Renal outcomes generally showed nephroprotective effects, with slower eGFR decline and lower AKI incidence (e.g., 30% lower AKI risk in HFpEF patients [12]). Quality of life data were limited in observational studies but likely improved due to reduced hospitalisations [16].

### 3.3. Safety Profile in Real‐World Use

Real‐world safety findings mirrored clinical trials, with no new adverse signals. Large analyses found no increased risk of amputations or severe hypoglycaemia; some even reported lower odds compared with other diabetes medications [16, 17]. Diabetic ketoacidosis (DKA), including rare cases of euglycaemic DKA, was rare, with slight increases noted in some studies, typically in insulin‐dependent patients with precipitants. Severe genitourinary infections such as Fournier′s gangrene were exceedingly rare but remain an important clinical consideration. Genitourinary infections were noted as a class effect, generally manageable. No adverse effects on kidney function emerged beyond the known initial eGFR dip. Overall, SGLT2 inhibitors were safe and well tolerated in broad HF populations [18].

## 4. Discussion

This meta‐analysis confirms that SGLT2 inhibitors significantly improve outcomes in HF patients across all ejection fractions and clinical profiles in real‐world settings, mirroring clinical trial benefits. Patients initiated on SGLT2 inhibitors experienced approximately a one‐third reduction in worsening HF events and death. This reproducibility in typical healthcare settings, even in older and more comorbid patients, underscores the broad effectiveness of SGLT2 inhibitors, including in HFpEF, a previously hard to treat phenotype [19–21]. The absolute benefits of SGLT2 inhibitors are clinically meaningful. For every 100 HF patients treated for about 1 year, approximately 1.17 fewer HF hospitalisations and several fewer deaths can be expected. The early onset of benefit, with risk curves diverging within weeks, suggests that SGLT2 inhibitors should be initiated as soon as feasible, potentially during or immediately after hospitalisation for acute decompensated HF. The broad applicability of these benefits, regardless of diabetes status or age, highlights their potential to improve both quantity and quality of life by keeping patients out of the hospital [22]. The striking concordance between real‐world effectiveness and randomised trial efficacy (e.g., HRs for composite outcomes around 0.6–0.7) supports the notion that SGLT2 inhibitors′ benefits extend beyond idealised settings, though observational data cannot definitively confirm causality [1, 23]. However, the slightly larger mortality benefits observed in our pooled analysis compared with pivotal trials may partially reflect inherent biases in observational data, including residual confounding and indication bias, and should therefore be interpreted with appropriate caution. Real‐world data also suggest a significant survival benefit in HFpEF, which individual trials were not always powered to definitively show. SGLT2 inhibitors provide incremental benefit even on top of guideline directed medical therapy, cementing their role as a foundational therapy for HFrEF and a key evidence‐based therapy for HFpEF, also offering nephroprotection [24, 25]. Our analysis leverages a large aggregate sample size (millions of patients), enhancing power and generalisability across diverse health systems and demographics. Rigorous meta‐analytic techniques, prespecified subgroup analyses, and thorough bias assessments (ROBINS‐I, NOS) were employed, and low heterogeneity and consistent sensitivity analysis results strengthen confidence in our conclusions [26, 27].

## 5. Limitations

Inherent to observational research, potential for residual confounding exists, as patients receiving SGLT2 inhibitors may differ in unmeasured ways. Specifically, indication bias may be present if healthier patients or those with better access to care are more likely to be prescribed these agents, which could inflate the observed treatment benefits relative to RCT estimates. Furthermore, immortal‐time bias could inflate observed benefits if the time between cohort entry and treatment initiation is misclassified as treated time; although several included studies employed active comparator designs and new‐user approaches to mitigate this, residual susceptibility cannot be entirely excluded. Although rigorous adjustment techniques were used, causation cannot be definitively proven. Treatment selection bias is also possible. The relatively short follow‐up (6–36 months) in most studies might not capture long‐term adherence or benefits, and may be insufficient to fully assess long‐term mortality trends or to capture rare but serious safety events such as euglycaemic DKA and Fournier′s gangrene. Additionally, the grouping of HFmrEF patients with the HFpEF cohort in most primary studies prevented a dedicated subgroup analysis of this phenotype. Despite these limitations, the remarkable consistency with RCT findings strongly suggests that the observed benefits are largely attributable to SGLT2 inhibitors themselves.

## 6. Conclusion

This comprehensive real‐world meta‐analysis demonstrates that SGLT2 inhibitors confer significant benefits across diverse HF phenotypes. Across patients with HFrEF, HFmrEF, and HFpEF, SGLT2 inhibitor use was associated with a substantial reduction in HF hospitalisations and improved survival. The pooled evidence indicates a 35% relative risk reduction in HF hospitalisation and a notable decrease in all‐cause and cardiovascular mortality with SGLT2 inhibitors, compared with nonuse. These benefits were remarkably consistent across subgroups—including patients with reduced or preserved ejection fraction and those with or without diabetes—underscoring a class effect that extends to broad HF populations. Importantly, our real‐world findings mirror the magnitude of benefit observed in pivotal randomised trials, reinforcing that the trial‐proven efficacy of SGLT2 inhibitors translates well into routine clinical practice. The consistency between observational and trial data bolsters confidence in the generalisability of SGLT2 inhibitors′ effects beyond controlled research settings. Furthermore, no new safety signals emerged in the aggregated real‐world data, and the safety profile (risk of ketoacidosis, genitourinary infections, etc.) was in line with expectations from clinical trials. Overall, the evidence affirms that SGLT2 inhibitors meaningfully improve HF outcomes on a population level, reducing both hospitalisation burden and mortality across the spectrum of ejection fraction phenotypes. These findings strengthen the rationale for SGLT2 inhibitors as a cornerstone therapy in HF and highlight their robust effectiveness in everyday care, without venturing into specific treatment recommendations or policy directives.

## Author Contributions

M.E.A.E. conceived and designed the study, performed the data analysis, interpreted the results, and drafted the manuscript. Critical revision of the manuscript was done by A.A.A.

## Funding

No funding was received for this manuscript.

## Disclosure

The authors approve the final version of the manuscript.

## Ethics Statement

The authors have nothing to report.

## Consent

The authors have nothing to report.

## Conflicts of Interest

The authors declare no conflicts of interest.

## Supporting information


**Supporting Information** Additional supporting information can be found online in the Supporting Information section. Table S1: Diagnostic criteria and coding definitions for heart failure across the data sources underlying the included observational studies, including ejection fraction ascertainment methods and country of origin for each registry or administrative database. Data S1: PROSPERO protocol registration details (Registration Number: CRD420261356715) and disclosure of post hoc deviations from the registered protocol, in accordance with PRISMA 2020 reporting guidelines.

## Data Availability

All the data used in the study are available from the first and corresponding author on reasonable request.
