# Supplementary material for: Real‐World Effectiveness of SGLT2 Inhibitors Across Heart Failure Phenotypes: A Meta‐Analysis
Source: J Diabetes Res. 2026 May 13;2026:6584068. doi: 10.1155/jdr/6584068 (PMC13170705; doi:10.1155/jdr/6584068)
Supplement: Supplementary file 1 — Supporting Information Additional supporting information can be found online in the Supporting Information section. Table S1: Diagnostic criteria and coding definitions for heart failure across the data sources underlying the included observational studies, including ejection fraction ascertainment methods and country of origin for each registry or administrative database. Data S1: PROSPERO protocol registration details (Registration Number: CRD420261356715) and disclosure of post hoc deviations from the registered protocol, in accordance with PRISMA 2020 reporting guidelines. [file JDR-2026-6584068-s001.docx]

Supplementary Materials

# Supplementary Table 1. Diagnostic Criteria and Coding Definitions for Heart Failure Across Data Sources Used in Included Studies

This table summarises the diagnostic criteria and coding systems used to identify heart failure (HF) cohorts across the data sources underlying the included observational studies. Where multiple studies utilised the same registry or administrative database, definitions are presented at the data-source level.

| Study / Registry | Country / Region | HF Diagnostic Criteria / Codes | Ejection Fraction Ascertainment |
| --- | --- | --- | --- |
| CVD-REAL 3 / EMPRISE (Pooled) | Multinational | ICD-10 codes (I50.x) in primary or secondary discharge position | Not routinely captured; presumed mixed HF |
| French National Health Data System (SNDS) | France | ICD-10 codes (I50.x) for hospitalisation or chronic disease registration (ALD) | Not captured (administrative claims data) |
| US Medicare / Commercial Claims | United States | ICD-9 (428.x) or ICD-10 (I50.x) + ≥1 HF medication | Not captured (administrative claims data) |
| Swedish Heart Failure Registry (SwedeHF) | Sweden | Clinical diagnosis + entry into national quality registry | Echocardiography (LVEF <40%, 40–49%, ≥50%) |
| UK Clinical Practice Research Datalink (CPRD) | United Kingdom | Read codes for heart failure in primary care records | Free-text extraction or linked secondary care data |
| Taiwan National Health Insurance Database | Taiwan | ICD-9/ICD-10 codes + prescription of loop diuretics | Not captured (administrative claims data) |
| Korean National Health Insurance | South Korea | ICD-10 codes (I50.x) + outpatient visits or hospitalisation | Not captured (administrative claims data) |
| Japanese National Database (NDB) | Japan | ICD-10 codes (I50.x) + claims-based definitions | Not captured (administrative claims data) |
| Multinational Real-World HF Cohorts | Multiple regions | Combination of ICD coding, registry data, and clinical diagnosis | Variable; often not consistently reported |

# Supplementary Appendix: Protocol Registration and Deviations

## PROSPERO Registration

The systematic review and meta-analysis protocol was prospectively registered on the International Prospective Register of Systematic Reviews (PROSPERO) under registration number: CRD420261356715.

## Post-Hoc Protocol Deviations

• Grouping of HFmrEF and HFpEF: The protocol initially planned separate analysis of HFmrEF. Due to limited reporting in primary studies, HFmrEF (LVEF 40–49%) was grouped with HFpEF (LVEF ≥50%).

• Inclusion of Active-Comparator Studies: Although non-users were the primary comparator in the protocol, high-quality active-comparator studies (e.g., vs DPP-4 inhibitors or GLP-1 receptor agonists) were included to reduce confounding and immortal-time bias.

• Meta-Analytic Model Selection: A DerSimonian–Laird random-effects model was used as the primary approach. A sensitivity analysis using Restricted Maximum Likelihood (REML) was added to confirm robustness.
